# Virtual Energy Management for Physical Energy Savings in a Legged Robot Hopping on Granular Media

**DOI:** 10.3389/frobt.2021.740927

**Published:** 2021-12-21

**Authors:** Sonia F. Roberts, Daniel E. Koditschek

**Affiliations:** Electrical and Systems Engineering, University of Pennsylvania, Philadelphia, PA, United States

**Keywords:** legged, locomotion, energy, granular, sand, reactive

## Abstract

We discuss an *active damping* controller to reduce the energetic cost of a single step or jump of dynamic locomotion without changing the morphology of the robot. The active damping controller adds virtual damping to a virtual leg spring created by direct-drive motors through the robot’s leg linkage. The virtual damping added is proportional to the intrusion velocity of the robot’s foot, slowing the foot’s intrusion, and thus the rate at which energy is transferred to and dissipated by the ground. In this work, we use a combination of simulations and physical experiments in a controlled granular media bed with a single-leg robot to show that the active damping controller reduces the cost of transport compared with a naive compression-extension controller under various conditions.

## 1 Introduction

Most approaches to legged robot locomotion assume rigid ground contacts. However, most surfaces outside of the built environment are soft. This includes natural environments common on planet Earth, such as forests, fields, hillslopes, beaches, and deserts; disaster sites in the built environment, which may include landslides; and of course extraterrestrial environments such as the notoriously soft soil on Mars. Many of these environments, when stepped on by a legged robot, and can be modeled as *granular media*: a collection of rigid, macroscopic particles which together exhibit bulk behavior forces.

In addition to being unpredictably deformable, which can cause a robot to slip or fall, granular media is also highly dissipative. The reaction forces exerted by even dry sand with homogeneous particles are complex, and understanding the interaction between the robot’s foot and the ground requires a good understanding of the force response of the ground to the forces associated with locomotion. Granular media physicists and robophysicists have developed bulk-behavior models of dry, homogeneous granular media in response to vertical intrusion (Li et al., 2013; Aguilar and Goldman, 2016). From models like this, we know that the bulk behavior of granular media approximates a one-directional spring with quadratic damping. With damping this high, it is not surprising that granular media is difficult and energetically costly to locomote over.

As part of a collaboration with geoscientists studying erosion in natural environments, we regularly take legged robots acting as “field assistants” to deserts, and forested hillslopes (Roberts et al., 2014a; Roberts et al., 2014b; Qian et al., 2017; Wilson et al., 2021). The energetic cost of transport is a recurring issue on these field trips, resulting not only in reduced battery life for the robots but also dangerously overheating motors. However, deserts are surprisingly variable environments. On previous field trips to White Sands National Monument, the Jornada Long-Term Experimental Range, and the Tengger desert, we observed differences in compaction, and therefore force responses, on the order of half a robot body length (about 1/3 m) (Sherman et al., 2013; Barchyn et al., 2014; Roberts et al., 2014a; Roberts et al., 2014b; Qian et al., 2017). Even in the best-case scenario, where the granular media itself is not changing from step to step (which is not a guarantee in natural landscapes), the forces will differ greatly from step to step because of this range of compactions. We therefore aim to develop a robust, analytically tractable controller that is effective at reducing the energetic cost of transport for locomotion on granular media without a model of the specific media. That is, our controller should not require parametric knowledge of the bulk-force response of the specific media encountered by some particular foot placement.

Strategies for increasing the capabilities of legged robots on sand are often inspired by the approaches employed by desert-specialized animals, which rely on general principles of the force response of the sand to intrusion. For example, increasing the area of the intruding foot increases the effective stiffness of the ground by increasing the depth-dependent “stiffness”-like force (Aguilar and Goldman, 2016), leading to faster locomotion (Li et al., 2009; Qian et al., 2015; Kolvenbach et al., 2021). Applying two parallel intruders similar to the long toes found on the feet of desert-specializing lizards could increase the effective area of the “foot” created by increasing the number of grains that the intruding object interacts with, which also increases the force response of the ground (Pravin et al., 2020). We have already adapted a robot for locomotion in the desert using these empirically demonstrated methods of improving locomotion performance by widening its feet (Roberts et al., 2014a,b) based on the research indicating that a reduced foot pressure increases the forward speed of the robot (Qian et al., 2015). Similarly, the use of a universal granular jamming gripper (Brown et al., 2010) as a foot (Chopra et al., 2020) allows a robot to spread its foot out flat on granular media without losing grip on the small footholds it may encounter on other kinds of rough terrain.

Overall, increasing a robot’s locomotor capabilities using general principles of the force response of granular media to intrusion by objects with different areas is a robust and effective method. It works on all types of granular media without requiring parametric knowledge of the bulk-force response of the specific media. In addition, increasing the force response from the ground by changing the morphology of the foot effectively increases the forward speed of the robot, thus improving the locomotion capability of the robot. If the power consumption is similar with larger and smaller feet, this may decrease the energetic cost of transport as a result. However, it is not always desirable nor possible to substantially change the morphology of the robot in order to adapt it to locomotion on new terrain.

Other researchers have worked to increase a legged robot’s locomotor capabilities while walking on sand by changing the robot’s controller. Changing parameters of a six-legged robot’s gait was sufficient to increase its speed in granular media (Li et al., 2009), and using different gaits can increase the maximum angle of a dune slope that the robot can climb (Roberts et al., 2014b). For example, if the robot’s rear right and left legs move at different times, a large amount of the bodyweight of the robot is supported by one leg at a time and that leg may overheat. If instead the robot uses a gait in which the back legs move together, the legs may not overheat as quickly, making it possible for the robot to climb higher dunes at steeper angles. The stability of a robot’s climb up an inclined dune can be affected by how dynamic the gait is (Kolvenbach et al., 2021). While most of this work has focused on either steady-state locomotion or single jumps, there has also been some attention paid to efficiently stopping locomotion in sand without dissipating too much energy into the substrate (Lynch et al., 2020). However, none of this work directly addresses the cost of transport during locomotion.

Model-based control methods can be used to increase locomotor capabilities as well. Optimal control and Gaussian process methods have been used to generate motor trajectories that allow a robot to jump to a prescribed height after learning the ground properties over the course of a series of initial jumps (Chang et al., 2017, 2019). In comparison to open-loop control, adaptive compliance control improves the stability of a legged robot walking on quartz sand (Zhu and Jin, 2016). Whole-body control of a quadruped with an estimated ground model based on a linear spring and damper shows increased stability in comparison to whole-body control without the estimated model (Fahmi et al., 2020), even though the state estimation drifts quickly (Fahmi et al., 2021). Finally, a reinforcement learning-based controller for a quadruped which uses an 8-dimensional vector to model perturbations from the ground and elsewhere in the environment shows improved stability in comparison to the native controller shipped with the robot when tested on a natural sand environment (Kumar et al., 2021). However, all these projects focus on stability and achieving goals such as specified jump heights, and do not address cost of transport as a primary or even a secondary goal after robustness and stability of locomotion.

Our approach differs from these other bodies of work in three important respects: First, we use only changes to control and not to robot morphology in order to decrease the cost of transport; second, our robot controller does not use a model of the granular media to achieve its results; and third, previous work on controllers for legged locomotion on granular media focuses on problems like maintaining a trajectory or achieving a prescribed jump height. In contrast, our work seeks to lower the energetic cost of a single jump as the primary goal, with a secondary goal of not reducing the robot’s apex height while jumping.

In previous work (Roberts and Koditschek, 2018; Roberts and Koditschek, 2019) we introduced a reactive controller for sand locomotion that uses a new variant of active damping to reduce the energetic cost of transport (see Section 2.2) and performed initial tests using a one-legged robot in simulation and emulation. In Roberts and Koditschek (2018) we introduced the reactive controller and showed in simulation that it reduced the energy necessary to jump on granular media relative to its base controller when varying forces from the ground and initial conditions. In Roberts and Koditschek (2019) we built a robotic platform that emulates the forces exerted by a simplified granular media model and tested a physical robot jumping with the active damping controller, varying the robot’s jump height. This paper addresses the potential benefit of the active damping controller for legged locomotion by further mathematical analysis and the first experiments on real, physical granular media rather than a simulation or a simplified emulation.

**FIGURE 1:**
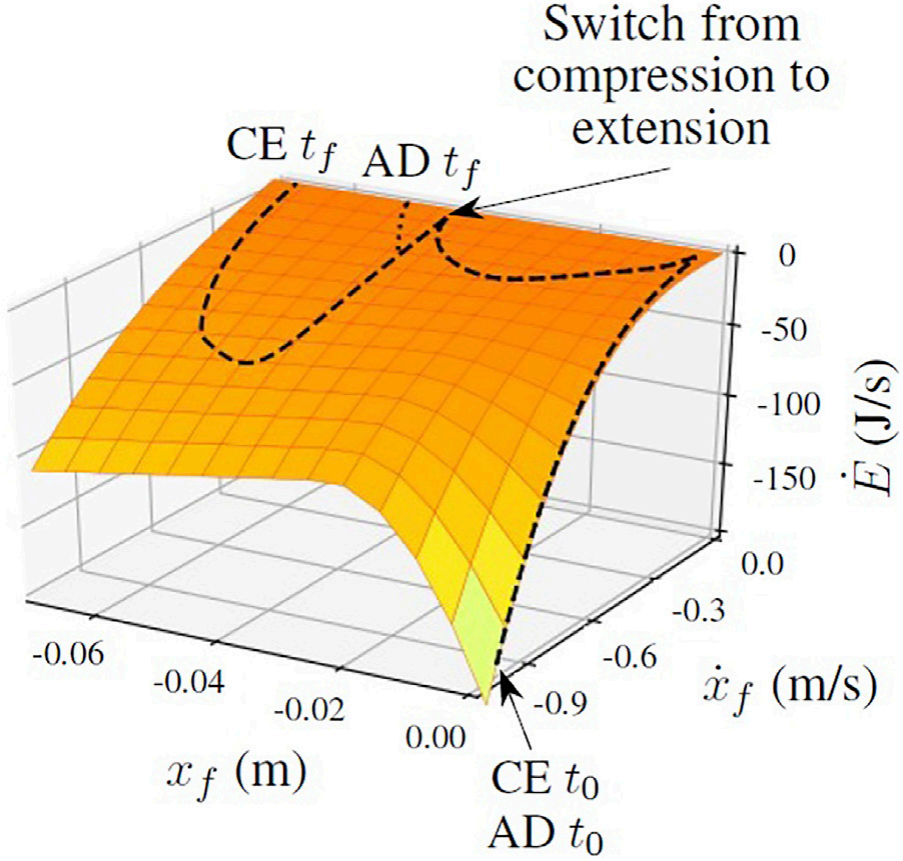
The compression-extension controller transfers more energy to the ground than the active damping controller during the extension mode. The surface indicates the rate of energy transfer between the robot’s foot and the ground as a function of the state of the foot, with lighter colors (yellow) indicating more energy transfer and darker colors (orange) indicating less energy transfer. It is the right side of Eq. 2, the power function of state associated with the total energy function. The dashed line plots a typical trajectory of the foot through state space when jumping using the compression-extension controller, while the dotted line plots the trajectory of the same foot from the same initial conditions using the active damping controller. The labels *t*_0_ and *t*_*f*_ refer to the initial and final timesteps of the trajectory. The lines overlap during the compression mode and only diverge during the extension mode, when the active damping controller is active. See Sections 2.2, 2.2.2 for more information.

**FIGURE 2:**
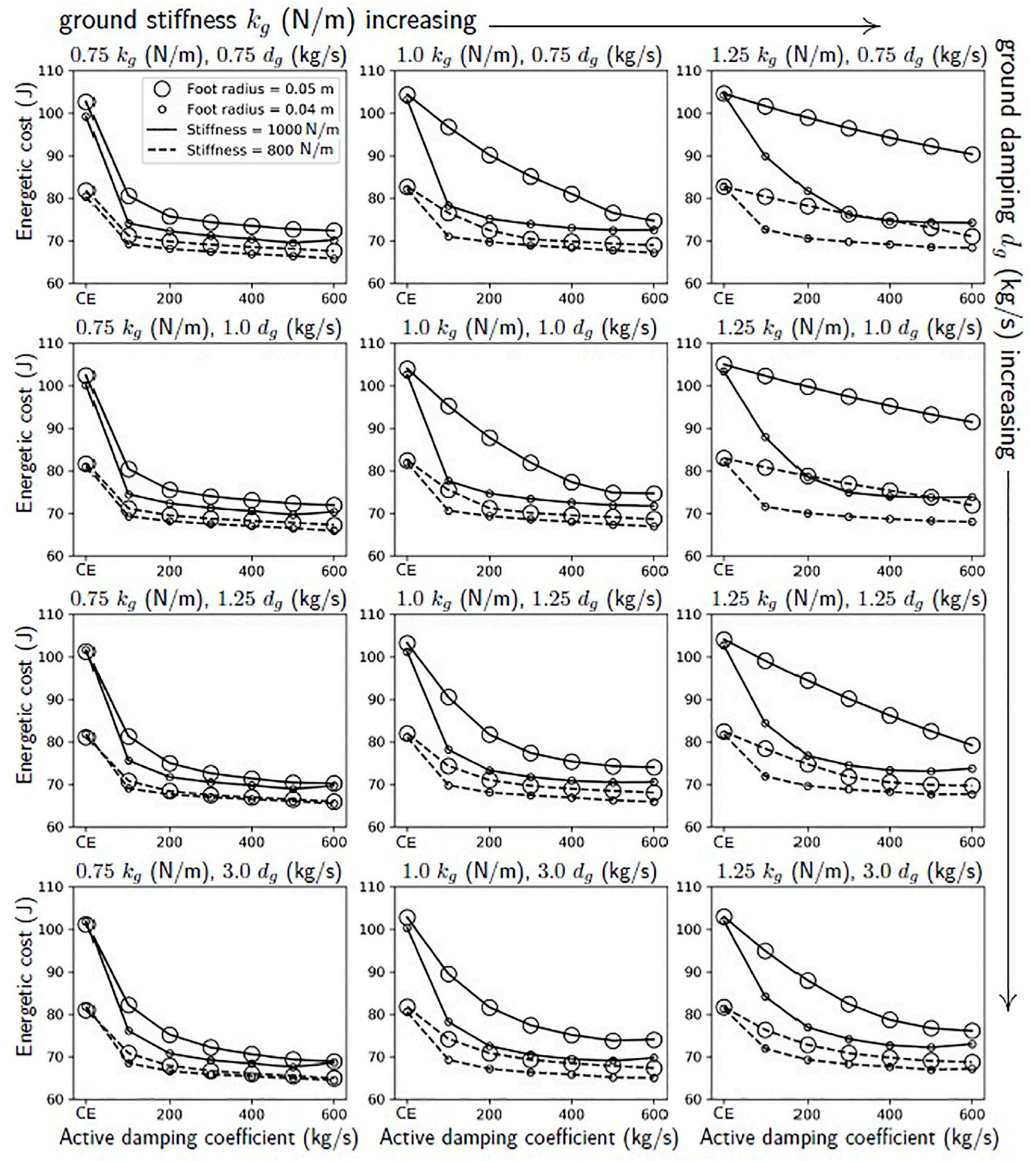
From simulations of the robot jumping on granular media, we expect that there should be a larger benefit to using active damping on a robot with a stiffer virtual extension spring and larger feet. These lines show the joules used in a simulation of a single jump with a range of active damping coefficients, foot sizes, extension stiffnesses, and scalings of the ground’s nominal stiffness and damping forces, *k*_*g*_ (N/m) and *d*_*g*_ (kg/s). These ground forces correspond to the stiffness and damping of fluidized, loosely packed poppyseeds. The points corresponding to the compression-extension controller are those for which the active damping coefficient equals zero. See Section 2.3.1 for more information.

**FIGURE 3:**
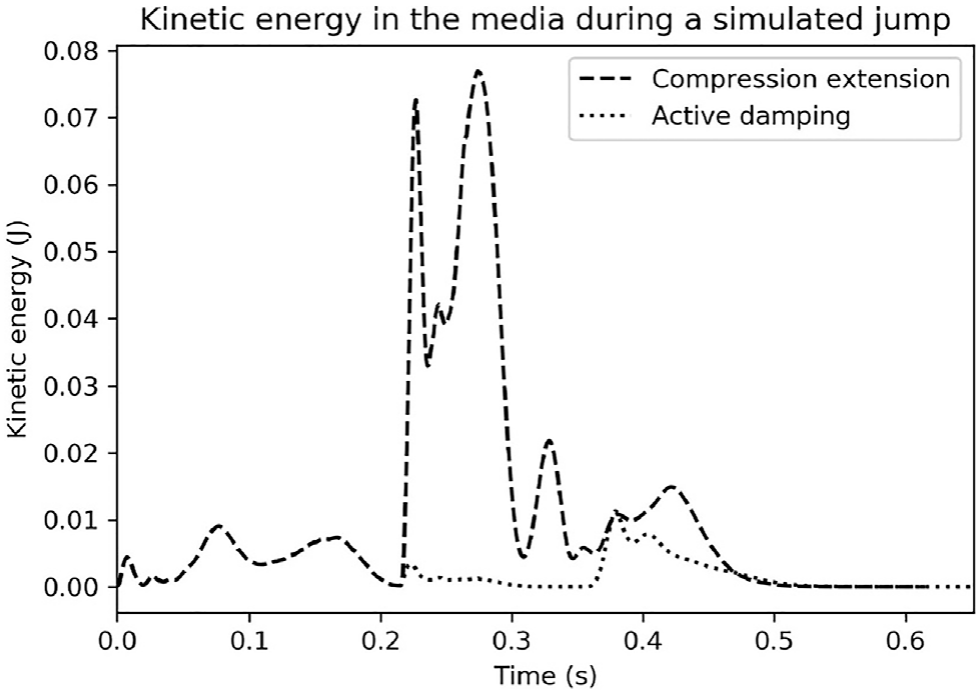
Discrete element model simulations run based on a trajectory produced in a simulation using the analytic force models. Notice how much more kinetic energy the ground absorbs under the compression-extension controller. See Sections 2.3.2, 2.2.2 for more information.

**FIGURE 4:**
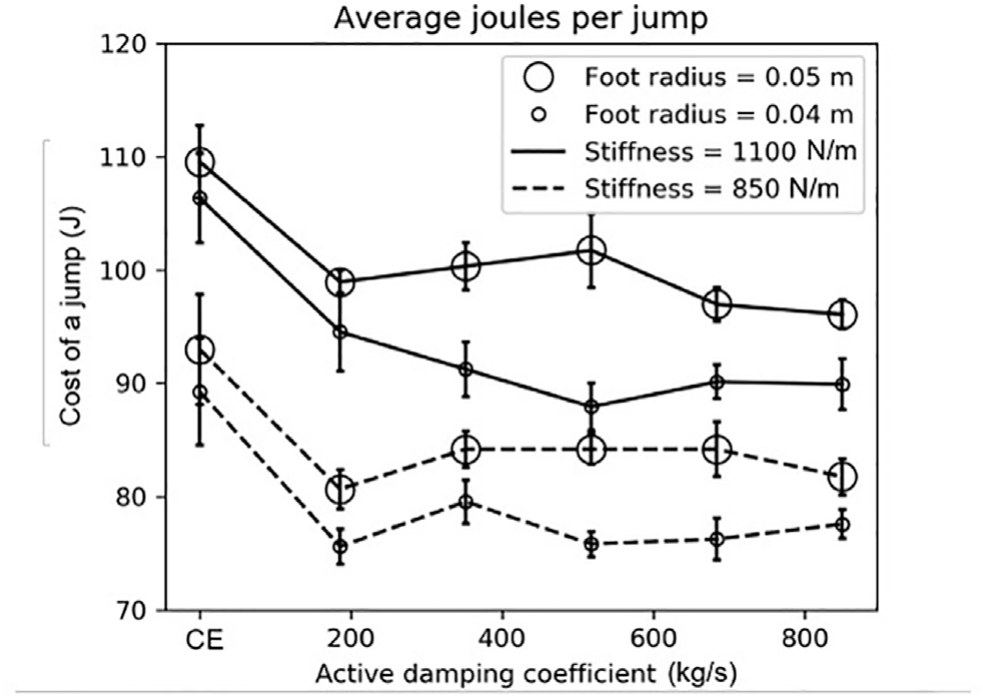
The robot used more energy with the compression-extension controller than the active damping controller when jumping on the granular media bed. Recall that the compression-extension controller corresponds to an active damping coefficient of zero. The foot radius had a larger effect on the joules per jump than the leg’s virtual spring stiffness during its extension mode. In this plot, the size of the circle indicates the foot’s radius, the line style indicates the stiffness gain during extension, and the horizontal bars indicate standard error. See Section 3 for more information.

**FIGURE 5:**
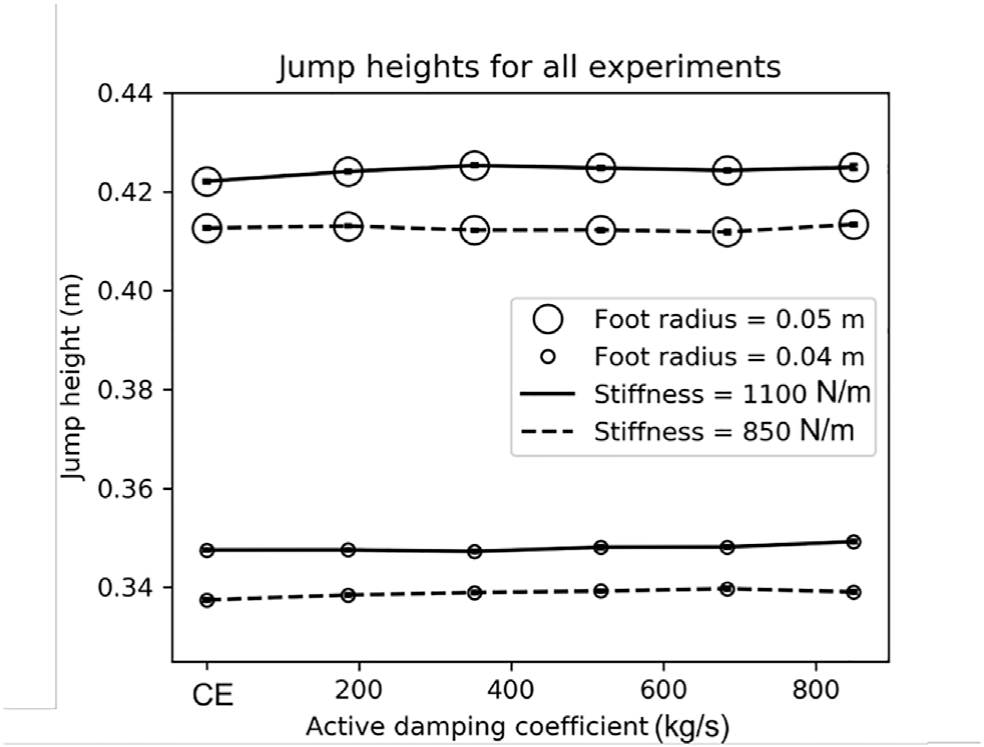
Using the active damping controller did not change the jump height. In most conditions, the robot jumped slightly higher (up to 3 mm) on average when using active damping. Open circles indicate the average height of the robot’s center of mass at the apex of the jump and horizontal lines indicate standard error. Line style indicates the leg spring’s stiffness during extension. See Section 3.4 for more information.

Specifically, the contributions of the present work are:

1) Mathematical analysis showing that the energy transferred to the ground when using active damping is strictly less than the energy transferred to the ground when using a comparison controller (Section 2.2.2; Figure 1)
2) Simulations comparing the effects of a wider range of parameters (foot size, extension stiffness, ground force functions, and active damping coefficient) than in Roberts and Koditschek (2018) and discrete element model simulations suggesting that active damping gives energetic savings for legged locomotion on sand (Section 2.3; Figures 2, 3)
3) Experiments on physical prepared granular media comparing the nominal and active damping controllers, corroborating the results of the simulations and analysis (Section 2.4; Figures 4, 5)

The rest of the paper is organized as follows. In Section 2.1 we describe the model of granular media used in this work, the quadrupedal robot we are targeting with this research, and the abstracted one-legged robot model that we used for simulations and instantiated in a physical robot in order to perform experiments on granular media. In Section 2.2 we discuss the controllers used on the robot. We first describe the standard compression-extension controller and then introduce the active damping controller (Section 2.2.1). We address Contribution 1 in Section 2.2.2 by using mathematical analysis to determine the conditions under which a one-legged robot jumping with the active damping controller should be expected to use less energy to jump than a robot jumping with the comparison controller. In Section 2.3 we describe two types of simulations that suggest that the active damping controller is more energetically efficient than the comparison controller (Sections 2.3.1, 2.3.2), addressing Contribution 2. Measuring and simulating the energy consumption shows that there is a difference between the nominal controller and the active damping controller, but does not provide an explanation for why such a difference exists. The analysis in Section 2.2.2 and the discrete element simulations in Section 2.3.2 offer an explanation: The nominal controller energizes the ground more than the active damping controller, thus dissipating more energy into the ground. Finally, in Section 2.4, we discuss the physical experiments. In Sections 2.4.1, 2.4.2 we describe the physical robot and the controlled granular media bed, respectively. The results from the physical experiments are in Section 3, addressing Contribution 3.

## 2 Materials and Methods

### 2.1 Target Systems and Models Used in This Study

In this section, we will first describe the analytical bulk-behavior force model of the sand. We will then describe the target quadrupedal robot and our one-legged model of the target robot.

#### 2.1.1 Analytical Force Model of Sand

The granular media bulk-behavior model used in these experiments (Aguilar and Goldman, 2016) was developed over many years by granular media physicists performing careful experiments on granular media with different properties. The model has three component forces. The first force *k*_*g*_ (*x*_*f*_), which is a function of depth *x*_*f*_ only, can be considered a “stiffness” function of the ground. This force comes directly from Resistive Force Theory (RFT) (Li et al., 2013), which describes the hydrostatic-like forces in response to vertical, angled, and or horizontal intrusion by an arbitrarily-shaped object^1^. The force response has two linear regions with a smooth transition. The first linear region has a higher stiffness, and this portion of the stiffness function corresponds to the recruitment period for the stagnant cone under the intruder. Larger intruders have a longer initial linear region than smaller intruders. The overall force response scales with the surface area of the intruding face, explaining why animals and robots with a larger foot area display better locomotion capabilities on dry, and homogeneous granular media (Qian et al., 2015).

The second force is the depth-dependent inertial drag term, $d_{g}\left(x_{f}\right){\dot{x}}_{f}^{2}$ . This force can be considered a quadratic “dissipation” function of the ground with a coefficient that changes with depth for small depths (Clark and Behringer, 2013; Bester and Behringer, 2017). As in the stiffness function, once the stagnant cone is fully formed, and the coefficient on the quadratic dissipation function becomes a constant. This term describes the hydrodynamic-like forces from intrusion into granular media. These forces result from the inertia of the grains accelerated to move along with the intruder, including both the grains that become part of the stagnant cone and the grains in the boundary layer next to the cone which are continuously recruited and shed from the fully-formed cone as the intruder pushes ever deeper.

The last force is the added mass force, $m_{a}\left(x_{f}\right){\ddot{x}}_{f}$ . This force describes the added mass of the stagnant cone to the intruding object. As with the other two forces, at low depths the recruitment of the grains into the cone of added mass creates a proportionately larger force as a function of depth. Once the stagnant cone is fully formed, the coefficient that is a function of depth levels off and increases only with the rate of continued shedding and recruitment of grains. The three forces together give the full model:

$$F\left(x_{f},{\dot{x}}_{f},{\ddot{x}}_{f}\right)=k_{g}\left(x_{f}\right)+\text{sign}\left({\dot{x}}_{f}\right)d_{g}\left(x_{f}\right){\dot{x}}_{f}^{2}+m_{a}\left(x_{f}\right){\ddot{x}}_{f}.$$ (1)

**FIGURE 6:**
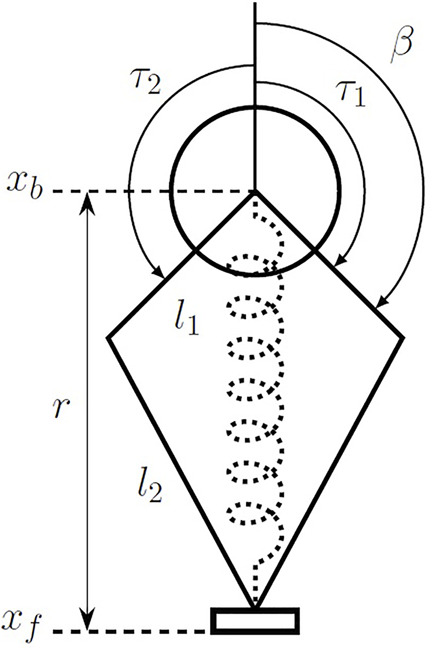
An abstracted model of the one-legged robot used in this study. This model was instantiated in simulation (described in Section 2.3.1) and as a physical robot (described in Section 2.4.1). The kinematics and inverse kinematics can be seen in Eq. 16. The two bars *l*_1_ and *l*_2_ are the symmetric leg linkages, with *r* the radius of the leg and *β* the angle of the first leg link. The positions of the body and foot in absolute terms are *x*_*b*_ and *x*_*f*_, respectively. The dotted spring in the middle of the figure denotes the virtual leg spring created by feedback control on the two motors operating in parallel in the body (at *x*_*b*_) through the leg linkage. The torques from each of the motors are denoted by *τ*_1_ and *τ*_2_.

Plots of the three force functions can be found in Roberts and Koditschek (2018) (Figure 6). An illustration of a robot foot intruding into granular media can be found in Aguilar and Goldman (2016), with a cone of recruited grains forming under the robot’s foot (Figure 2). Note that the term $\text{sign}\left({\dot{x}}_{f}\right)$ is required in order to make the damping force act in the correct direction when the foot pushes into the ground with a negative velocity.

#### 2.1.2 Description of Quadrupedal Robot Targeted With This Research

**FIGURE 7:**
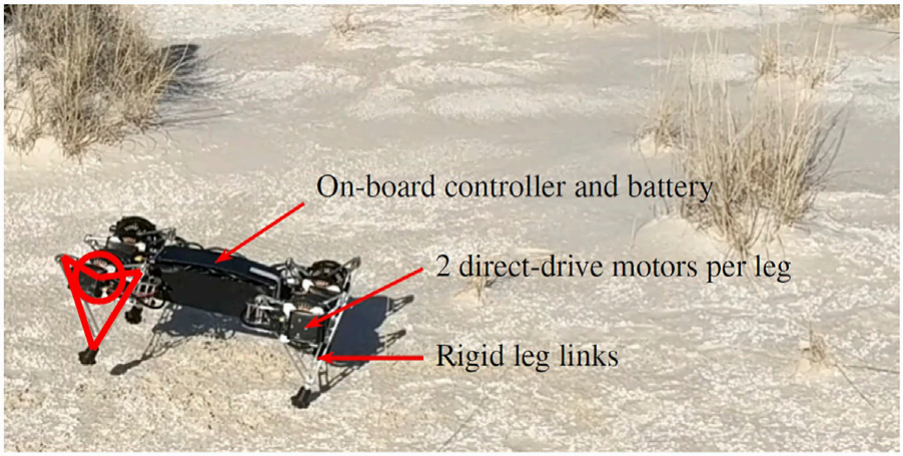
The Ghost Minitaur robot running in White Sands National Monument (New Mexica, United States), a common study site for geoscientists. This compacted, flat interdune area was the only flat area of the desert where the robot could reliably run. It was also able to run downhill on compacted dune faces. The robot’s legs consist of two direct-drive motors in parallel which drive opposing symmetric leg linkages. The leg links are rigid, but the proportional-derivative control on the motors through the kinematics of the leg linkage makes the overall behavior of the legs like damped springs. The markings on the robot’s front left leg (left side of the image) show the correspondence between the diagrammatic figures which appear later in the paper and the physical quadrupedal robot. In this photo, the robot is *bounding*: The front two legs and back two legs are paired. See the Supplementary Video for a video clip of the robot bounding.

The Minitaur^2^ (Kenneally et al., 2016) robot (Figure 7) is a quadrupedal robot with direct-drive legs (no gearboxes). All of the robot’s components are rigid, but the legs exert spring-like forces using feedback control through their linkages. The legs therefore behave like *virtual springs*, with a virtual stiffness coefficient, and damper that can be changed programmatically to control locomotion. Because the forces from the legs are known, when they are compressed by environmental perturbations, they can be used as force sensors, and provide haptic feedback to the robot (Kenneally et al., 2016; Topping et al., 2019). This is referred to as “direct-drive transparency” and provides great benefit to using a direct-drive architecture.

Minitaur nominally uses a variant (De and Koditschek, 2018) of *active damping* control (Seçer and Saranlı, 2013) to stabilize its vertical (hopping) degree of freedom. Here, “active damping” refers to a type of controller that actively changes the damping coefficient of a virtual spring to affect the behavior of a system. Minitaur’s nominal vertical hopping controller works in the following manner (De and Koditschek, 2018). First, the leg detects touchdown using a position error on its leg length. Then, while maintaining one virtual stiffness, the leg’s virtual damper sweeps through a sinusoidal curve that dips below zero as the leg finishes contracting and begins to expand. The negative virtual damping injects energy into the physical system by causing the actuators to apply greater torque, in turn causing the robot to expand its leg more quickly and jump.

By pairing legs, Minitaur can use a series of virtual bipedal gaits: Trotting, bounding, and or pronking. For example, a forward bounding gait would have the two front legs and the back legs paired, creating a virtual biped gait with one front leg, and one rear leg. To trot, the front right and rear left legs would be paired, as would the front left and rear right legs. When two legs are paired together into one virtual pair, the positions and velocities of the individual legs, and thus the forces to exert, are averaged to produce the length, and velocity of the virtual leg.

The overall control of the robot is typically a composition of virtual spring- and damper-like forces, which can result in a variety of robust steady-state and transitional locomotion behaviors (De and Koditschek, 2015; Topping et al., 2019). In our example of a composition of controllers for forward bounding, the robot would at bare minimum have the following controllers. First, it would have a controller for vertical hopping on each of the virtual legs. When triggered, this controller would inject energy into the physical legs, causing the pair of legs comprising each virtual leg to jump. Second, the robot would have a controller for how the legs sweep backward during stance to affect the forward speed. If this controller is set to a neutral position, the robot would hop up and down on its front and back pairs of legs, and bounding in place. If this controller is set to a forward position, the robot would move its legs backwards during stance so that it pushes itself forward as well as up during each jump. Finally, the robot would have a controller for roll that keeps the body level by increasing or decreasing the length of the legs on that side of the robot’s body in response to IMU data. Each of these controllers would operate in parallel to exert spring- and damper-like forces to keep the robot moving forward in steady state. More controllers can be added to increase stability and robustness, such as a controller for the yaw direction and a controller for which direction the robot considers “up” since the morphology is reversible. This composition is the robot’s fastest gait, and it has been shown to be robust to perturbations (De and Koditschek, 2018). The quadrupedal robot can be seen bounding down a dune in the Supplementary Video.

#### 2.1.3 Description of the Abstracted One-Legged Robot Model Used in This Study

In the present work, we address Minitaur’s vertical hopping controller with a one-dimensional one-legged model jumping on granular media. The model is instantiated in simulation (see Section 2.3.1) and in the physical world (see Section 2.4.1) with a one-legged robot attached to a linear rail and jumping in a box with granular media. An abstracted model of the robot which applies to both simulation and the physical robot can be seen in Figure 6. The robot has two motors in the body which operate in parallel. Though they rotate in opposite directions, since the robot is constrained to move only vertically, and the forces from the motors should always be equal.

We chose not to use Minitaur’s typical vertical hopping controller as our base controller in this study for two reasons. The first reason was that when we took the robot to White Sands National Monument in 2016, we had great difficulty in getting the robot’s legs to trigger touchdown appropriately. This is essentially a gain-tuning issue, with different gains being required for different ground stiffnesses, and it would almost certainly be possible to adjust the existing vertical hopping controller to jump on soft ground. However, since we aimed to develop a robust vertical hopping controller that did not require a model of the environment, we wanted to avoid adding anything to our controller that required modeling the ground stiffness. The second, and less important reason, was that Minitaur’s typical vertical hopping controller uses negative damping to energize the leg, with the consequence that the foot moves at high velocity during stance. Since the dissipation function of the ground is quadratic with velocity, this high-speed interaction with the ground is bound to dissipate a lot of energy quickly. We instead modeled the vertical hopping controller using a simple compression-extension controller (described in the next section), a well studied and robust type of control for dynamic legged locomotion.

### 2.2 Description and Analysis of the Compression-Extension and Active Damping Controllers Used in This Study

In this section, we first describe the compression-extension (base) controller and the active damping (experimental) controller. We then describe the analysis we performed to determine the conditions under which the active damping controller should be expected to expend less energy than the compression-extension controller.

#### 2.2.1 Description of the Compression-Extension and Active Damping Controllers

A “compression-extension” controller uses a soft virtual leg spring during the first half of stance, when the leg is compressing, and then injects energy into the leg spring using some form of active impedance control during the second half of stance, when the leg is extending. Increasing the energy in the leg spring when switching between the compression, and extension modes pushes the body up and the robot jumps. Active impedance controllers have been around for decades (Raibert, 1986; Hurst et al., 2004; Semini et al., 2015; Kenneally et al., 2016) and are sometimes used to support controllers developed through other means to increase the robustness of the locomotion behavior overall (Park et al., 2017).

The specific compression-extension controller used on our robot in this study operates in the following manner. The robot’s leg is programmed to hold the same nominal leg length throughout the duration of the jump. There are two virtual stiffness gains: A soft gain for the compression mode, and a stiff gain for the extension mode. The soft gain is sufficiently soft that the robot’s leg compresses easily under the weight of the body, and the stiff gain is sufficiently stiff that the robot leg expands with great force and pushes the robot’s body up very quickly, causing the robot to jump. In the robot used for these experiments, the virtual leg spring is created by the direct-drive motors through the kinematics of the leg linkage.

**FIGURE 8:**
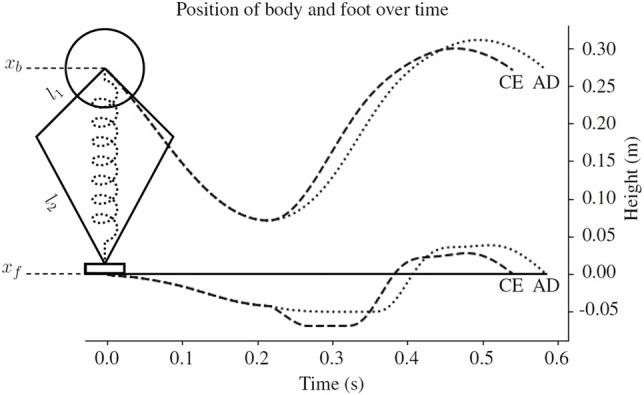
An example body and foot trajectory from the simulated robot using the compression-extension (CE, dashed line) and active damping (AD, dotted line) controllers described in Section 2.2. In the simulation, *l*_1_ = 0.1 m and *l*_2_ = 0.2 m. The neutral leg length, the distance between the body center of mass (*x*_*b*_) and the foot (*x*_*f*_), is 0.27 m. When the virtual leg spring switches from a soft compression gain to a stiff extension gain, the compression-extension controller pushes the foot further into the ground. The active damping controller does not. See Section 2.2 for more information.

On soft ground like sand, stiffening the virtual leg spring does not only push the body up, and but also quickly pushes the robot’s foot into the ground (Figure 8). A clip of the simulated robot jumping using on granular media is also included in the Supplementary Video. Because sand is highly dissipative, moving the foot further into the ground at high velocity results in a large loss of energy. In Figure 1, the surface indicates the rate at which energy is transferred between the foot and the ground as a function of the state of the foot. Let us consider the rate at which energy is absorbed by the ground.

First, notice that the energy used to plastically deform the ground cannot be recovered because the ground does not exert restoring forces. The spring potential energy $\int_{0}^{x_{f}}k_{g}\left(x_{f}\right)dx_{f}$ therefore represents the first mode by which the robot’s foot transfers energy to the ground. The rate of change of this energy with time is ${\dot{E}}_{spring}=\frac{d}{dt}\int_{0}^{x_{f}}k_{g}\left(x_{f}\right)dx_{f}=k_{g}\left(x_{f}\right){\dot{x}}_{f}$ . This is then the rate at which the ground is gaining “spring potential energy.” Next, notice that the uni-directional “spring” formed by the foot-ground system has a dissipation function, meaning that we can treat the dissipation of its energy to the ground’s “damper” like we would any other spring-mass system with a damper. For a typical spring-mass damper system with a damper that is linear with velocity $d_{lin}{\dot{x}}_{f}$ , recall that the rate at which the damper dissipates energy works out to $d_{lin}{\dot{x}}_{f}\cdot {\dot{x}}_{f}=d_{lin}{\dot{x}}_{f}^{2}$ . For a quadratic damper $d_{quad}{\dot{x}}_{f}^{2}$ , the rate at which energy is dissipated is cubic with velocity rather than quadratic. In order to be able to consider motion in the negative direction, we must add an absolute value sign to ensure that the energy dissipated is never negative, which would imply that energy is *gained* by the system: $d_{quad}|{\dot{x}}_{f}^{3}|$ . For our system with quadratic dissipation function $d_{g}\left(x_{f}\right){\dot{x}}_{f}^{2}$ , the rate at which energy is dissipated by the ground is therefore $d_{g}\left(x_{f}\right)|{\dot{x}}_{f}^{3}|$ . The rate at which energy is lost to the ground can then be written as

$$\dot{E}=-k_{g}\left(x_{f}\right){\dot{x}}_{f}-d_{g}\left(x_{f}\right)|{\dot{x}}_{f}^{3}|.$$ (2)

This equation describes the “power function” of the ground, or the rate at which energy is physically transferred from the foot to the ground. See Roberts and Koditschek (2018) for a more detailed derivation.

Notice that we do not include the mass function *m*_*a*_ (*x*_*f*_) in the power function. We decided not to include this function for several reasons. First, the kinetic energy of the mass of grains at the time when the robot lifts off is zero no matter what controller the robot is using, meaning that all of the kinetic energy in the ground subsystem is taken up either into the spring potential function or dissipated by the damping function. Second, the forces produced by the added mass function are much smaller than the forces produced by either the stiffness or damping functions of the ground. In previous simulation work, we found that the forces from the added mass function were two orders of magnitude smaller than the forces from the stiffness or damping functions (Roberts and Koditschek, 2018). There might be a different amount of potential energy in the foot depending on whether the cone of added mass under the robot’s foot is fully formed or whether the robot has penetrated the ground further using one controller, but in our previous simulation work this difference in potential energy was negligible compared to the energy taken up by the ground’s stiffness function and dissipated by its damper (Roberts and Koditschek, 2018).

Figure 1 shows the surface defined by this power function with two trajectories through the foot’s state space during stance. If a trajectory stays in the upper regions of the surface, the foot is transferring very little energy to the ground. The foot starts with a large negative velocity and at depth 0, but quickly slows its velocity as the soft leg spring compresses. The leg continues to compress as the whole robot sinks into the sand, causing the first dip along the *x*_*f*_ axis. Once the leg reaches its maximum compression and starts to extend, the robot switches from the soft spring of compression mode to the stiff spring of extension mode. This causes the second dip along the *x*_*f*_ axis: Stiffening the leg pushes the small foot further into the ground as it also pushes the body up.

In (Roberts and Koditschek, 2018), we introduced the *active damping* controller. The active damping controller adds virtual damping to the virtual leg spring in proportion to the intrusion velocity of the foot during the extension mode (Figure 3). This is accomplished by adding a new force term, *F*_*AD*_:

$$F_{AD}=d_{AD}|{\dot{x}}_{f}|\left({\dot{x}}_{b}-{\dot{x}}_{f}\right),$$ (3)

where *d*_*AD*_ is the active damping gain and ${\dot{x}}_{f}$ , ${\dot{x}}_{b}$ are the velocities of the foot and body, respectively. This force only acts when the foot’s intrusion velocity is negative. This one force during extension (Figure 4) reduces the energy transferred from the robot’s battery through its leg linkage to its foot and thus into the ground. Notice that in contrast to the form of active damping used in Minitaur’s nominal controller (Section 2.1.2), this controller adds *positive* damping rather than negative.

#### 2.2.2 Analysis of Active Damping Controller

To determine when the active damping controller should cost less energy than the compression-extension controller to perform a single jump on granular media, we compared the rate at which energy is transferred from the foot to the ground (Eq. 2) under the two controllers. Recall that *k*_*l*_ and *d*_*l*_ are the stiffness and damping coefficients of the virtual leg spring, *k*_*g*_ and *d*_*g*_ are the stiffness and damping coefficients of the ground, *m*_*b*_ and *m*_*f*_ are the masses of the robot’s body and foot, and *x*_*b*_ and *x*_*f*_ are the positions of the robot’s body and foot. Finally, *l* is the nominal length of the robot’s leg spring and *g* is the gravitational constant. Recall also that the ground exhibits no restoring forces and has quadratic damping. For the purposes of this analysis, assume that the ground stiffness is linear and the ground damping is constant in depth, *k*_*g*_ (*x*_*f*_) = *k*_*g*_ *x*_*f*_ and $\text{sign}\left({\dot{x}}_{f}\right)d_{g}\left(x_{f}\right){\dot{x}}_{f}^{2}=\text{sign}\left({\dot{x}}_{f}\right)d_{g}{\dot{x}}_{f}^{2}$ . The equation of motion for the robot’s foot under the compression-extension controller is then

$$m_{f}{\ddot{x}}_{f}=k_{l}\left(x_{b}-x_{f}-l\right)+d_{l}\left({\dot{x}}_{b}-{\dot{x}}_{f}\right)-k_{g}x_{f}-\text{sign}\left({\dot{x}}_{f}\right)d_{g}{\dot{x}}_{f}^{2}-m_{f}g.$$ (4)

Recall that the active damping force from Eq. 3 is added to the leg damping. Thus, the equation of motion for the robot’s foot under the active damping controller is

$$m_{f}{\ddot{x}}_{f}=k_{l}\left(x_{b}-x_{f}-l\right)+\left(d_{l}+d_{AD}|{\dot{x}}_{f}|\right)\left({\dot{x}}_{b}-{\dot{x}}_{f}\right)-k_{g}x_{f}-\text{sign}\left({\dot{x}}_{f}\right)d_{g}{\dot{x}}_{f}^{2}-m_{f}g.$$ (5)

Next, we solve each of these equations for *x*_*f*_. For $x_{f}^{CE}$ the depth of the foot under the compression-extension controller, this gives us

$$x_{f}^{CE}=\frac{+d_{l}{\dot{x}}_{b}-d_{l}{\dot{x}}_{f}-\text{sign}\left({\dot{x}}_{f}\right)d_{g}{\dot{x}}_{f}^{2}-lk_{l}-{\ddot{x}}_{f}m_{f}+k_{l}x_{b}-m_{f}g}{k_{g}+k_{l}}$$ (6)

and for $x_{f}^{AD}$ the depth of the foot under the active damping controller, this gives us

$$x_{f}^{AD}=\frac{+d_{AD}{\dot{x}}_{b}|{\dot{x}}_{f}|+d_{l}{\dot{x}}_{b}-d_{AD}|{\dot{x}}_{f}|{\dot{x}}_{f}-d_{l}{\dot{x}}_{f}-\text{sign}\left({\dot{x}}_{f}\right)d_{g}{\dot{x}}_{f}^{2}-lk_{l}-{\ddot{x}}_{f}m_{f}+k_{l}x_{b}-m_{f}g}{k_{g}+k_{l}}.$$ (7)

Recall the power function of the ground (Eq. 2) is $\dot{E}=-k_{g}\left(x_{f}\right){\dot{x}}_{f}-d_{g}\left(x_{f}\right)|{\dot{x}}_{f}^{3}|$ . We can now substitute in the values for $x_{f}^{CE}$ and $x_{f}^{AD}$ to get the power functions under the compression-extension and active damping controllers. For *P*_*CE*_ the power function of the ground under the compression-extension controller, this simplifies to

$$P_{CE}=-d_{g}|{\dot{x}}_{f}^{3}|+\frac{{\dot{x}}_{f}k_{g}\left(d_{l}{\dot{x}}_{f}-d_{l}{\dot{x}}_{b}+\text{sign}\left({\dot{x}}_{f}\right)d_{g}{\dot{x}}_{f}^{2}+lk_{l}+m_{f}g+{\ddot{x}}_{f}m_{f}-k_{l}x_{b}\right)}{k_{g}+k_{l}}$$ (8)

and for *P*_*AD*_ the power of the active damping controller, this simplifies to

$$P_{AD}=-|{\dot{x}}_{f}^{3}|d_{g}+\frac{{\dot{x}}_{f}k_{g}\left(|{\dot{x}}_{f}|d_{AD}+d_{l}\right)\left(-{\dot{x}}_{b}+{\dot{x}}_{f}\right)+{\dot{x}}_{f}k_{g}\left(\text{sign}\left({\dot{x}}_{f}\right)d_{g}{\dot{x}}_{f}^{2}+lk_{l}+m_{f}\left(g+{\ddot{x}}_{f}\right)-k_{l}x_{b}\right)}{k_{g}+k_{l}}$$ (9)

Notice from the formulation of Eq. 2 that *P*_*AD*_ and *P*_*CE*_ are both always negative. To find out under which conditions *P*_*CE*_ has the larger magnitude, we can subtract *P*_*CE*_ from *P*_*AD*_ and look for the conditions under which this quantity is positive. The difference simplifies down to

$$P_{AD}-P_{CE}=-\frac{|{\dot{x}}_{f}|{\dot{x}}_{f}d_{AD}\left({\dot{x}}_{b}-{\dot{x}}_{f}\right)k_{g}}{k_{g}+k_{l}}.$$ (10)

Since $\frac{k_{g}}{k_{g}+k_{l}}$ is a constant (the ground stiffness over the sum of the ground and leg stiffnesses), we can divide it out. Simplifying this slightly, we are left with

$$P_{AD}-P_{CE}\propto -\text{sign}\left({\dot{x}}_{f}\right){\dot{x}}_{f}^{2}d_{AD}\left({\dot{x}}_{b}-{\dot{x}}_{f}\right).$$ (11)

Assuming negative foot velocity ${\dot{x}}_{f}$ and positive active damping gain *d*_*AD*_, this quantity is greater than zero when ${\dot{x}}_{f}<{\dot{x}}_{b}$ . When the foot velocity and active damping gain are both negative, this quantity is greater than zero when the body and foot velocity are both negative, and the body is moving faster than the foot ( ${\dot{x}}_{b}<{\dot{x}}_{f}<0$ ).

The first situation could occur when the foot accelerates more quickly than the body in response to a change in virtual leg stiffness—for example, in a robot with more mass in its body than its foot switching from a soft compression gain to a stiff extension gain when the leg is fully compressed. The second situation could occur when the robot’s body accelerates more quickly than its foot, for example if the foot mass is greater than the body mass, and or (more likely) if the ground is very stiff. Under these conditions, adding energy to the leg with a negative velocity gain—as in the standard Ghost Minitaur bounding gait—will result in more energetically efficient locomotion than a naive compression-extension controller. However, on compliant ground, adding damping to reduce the foot’s intrusion velocity saves energy.

### 2.3 Simulations

In this section, we will describe the simulation experiments we performed. First, we will describe a series of simulations of single jumps using the analytical force models of granular media described in Section 2.1.1. In these simulations, we estimated the energy consumption of the robot as it jumped on granular media that exerts forces according to the analytic force model of the ground. Then, we will describe two discrete element model simulations using trajectories generated by the analytic force model simulations. The DEM simulations show how the kinetic energy of the ground changes under the two controllers. These simulations show a potential mechanism for how the active damping controller may be losing less energy to the ground than the compression-extension controller.

#### 2.3.1 Simulations Using the Analytic Force Models

We performed a series of simulations on idealized granular media using the force functions from the added mass model (Aguilar and Goldman, 2016) described in Section 2.1.1 to determine the effect of changing the foot size, the virtual extension stiffness, and the active damping coefficient^3^ The simulation program was custom-built in Python using Euler’s method to simulate the robot-ground interaction as a discrete dynamical system with a very small time step (dt = 10^–6 s^) and based on the program used to produce previous results on locomotion on granular media (Roberts and Koditschek, 2018). The simulation code is available on GitHub (see Data Availability Statement) and an example of the compression-extension controller and the active damping controller can both be seen in the Supplementary Video. Whereas in Roberts and Koditschek (2018) we compared the effects of initial velocity and scaled the forces from the ground, in these simulations we compare the effects of scaling the ground forces, the extension stiffness, the size of the foot, and the active damping coefficient. We used coefficients for the ground forces that were measured for poppyseeds (Li et al., 2013), the same linear scaling parameter as in (Hubicki et al., 2016), and as in (Roberts and Koditschek, 2018), we set the integration constant *C* equal to 1. The physical meaning of this constant in the model is the rate of shedding and re-recruiting of grains under the robot’s foot as it continues to move through the granular media after the cone of added mass is fully formed.

The simulated robot was assumed to have a mass of 1.75 kg in its body and 0.175 kg in its foot (10*%* of body mass), and was constrained to move vertically (Figure 3). A programmable linear spring connected the body and foot masses with an update loop of 1 kHz, the same as the update loop of the physical robot. The leg spring had a small damping coefficient, *d*_*l*_ = 0.5 kg/s during compression and *d*_*l*_ = 5 kg/s during extension. The nominal leg length was set to 0.27 m and the gravitational constant was set to 9.81. See Figure 3 for details.

The simulation had two modes: Stance and ballistic flight. During stance, the robot and the ground were modeled as a two-mass, two-spring system with nonlinear springs. With $x_{b}^{n},x_{f}^{n}$ the body and foot center of mass at timestep *n*, *k*_*l*_ and *d*_*l*_ the virtual leg stiffness and damping coefficients, *l* the neutral leg length, and *g* the gravitational constant, the force from the leg spring was as follows:

$$F_{bf}=k_{l}\left(x_{b}-x_{f}-l\right)+d_{l}\left({\dot{x}}_{b}-{\dot{x}}_{f}\right).$$ (12)

Let *m*_*b*_ be the mass of the body. Let *m*_*f*_ be the mass of the robot’s foot. Recall from Section 2.1.1 that the foot recruits mass as it moves through the ground, forming a cone underneath the robot’s foot. At timestep *n*, the mass of the foot may be different than at timestep *n* + 1. Therefore let $m_{f}^{n}=mf\left(x_{f}^{n}\right)$ be the mass of the foot at timestep *n*. Recall the stiffness and damping functions for the ground from Section 2.1.1, *k*_*g*_ (*x*_*f*_) and $\text{sign}\left({\dot{x}}_{f}\right)d_{g}\left(x_{f}\right){\dot{x}}_{f}^{2}$ . The forces from the ground at timestep *n* were therefore calculated as:

$$F_{g}^{n}=k_{g}\left(x_{f}^{n}\right)+\text{sign}\left({\dot{x}}_{f}^{n}\right)d_{g}\left(x_{f}^{n}\right){\left({\dot{x}}_{f}^{n}\right)}^{2}.$$ (13)

Then, the accelerations of the body and foot at time *n* in stance were calculated as:

$${\ddot{x}}_{b}^{n}=-\frac{F_{bf}}{m_{b}}-g$$ (14)

$${\ddot{x}}_{f}^{n}=\frac{F_{bf}}{m_{f}^{n}}-\frac{F_{g}}{m_{f}^{n}}-g$$ (15)

The switch from the robot’s compression spring stiffness to extension spring stiffness occurred when the following conditions were met: Either the leg length rate of change and the body velocity were both positive, ${\dot{x}}_{b}-{\dot{x}}_{f}>0\;\&\;{\dot{x}}_{b}>0$ , or the leg length was below a threshold, *x*_*b*_ − *x*_*f*_ < 0.105. That is, either the leg was starting to extend after having been compressed, or it had been compressed nearly as far as possible before reaching a singularity in the kinematics of the leg.

As the robot was extending its leg but before its foot lifted off of the ground, the forces the robot exerted on the ground and the forces from the ground were near equal. As a result, during this part of the simulation, the ground would switch between providing force and no force based on whether the current foot velocity was negative. If the foot velocity was negative, the ground would push back with the force determined by the depth, and velocity of the foot. If the foot velocity was positive or zero, the ground would give zero force. This prevented the ground from providing restoring forces and acting as a two-directional spring instead of a one-directional spring. When the robot’s leg first started to extend, the foot was pushed further into the ground. However, after the forces from the leg spring and the ground equalized, the foot’s velocity would switch between having a very small positive velocity, and having a very small negative velocity as the leg continued to extend. The ground therefore switched between providing force and providing no force with each timestep during the last part of each stance mode. We post-processed the simulation logs, in which we recorded the forces from the ground and robot, to replace the zero force values that occurred before lift-off. Lift-off was determined to occur when the forces from the leg were greater than $m_{f}^{n}\cdot g$ . During flight mode, the simulation dropped the added mass from the robot’s foot and set the ground forces to zero to avoid restoring forces. We simulated the body and foot mass in ballistic flight while connected by a spring.

We calculated the “cost to jump” in simulation using a simple motor model and the inverse kinematics of the robot’s leg. For *l*_1_, *l*_2_ the lengths of the robot’s leg linkages, *r* the length from the robot’s foot to the motor center of mass, and *β* the angle of the first link relative to the vertical, the inverse kinematics are:

$$\theta =\pi -\beta ={\text{cos}}^{-1}\left(\frac{l_{1}^{2}-l_{2}^{2}+r^{2}}{2l_{1}r}\right).$$ (16)

For *Df*|*r* the transpose Jacobian taking forces *f* from the end effector at leg radius *r* through the linkage to the torque at the motor, the torque for the leg spring force *F*_*bf*_ is then *τ* = *Df*|_*r*_ *F*_*bf*_. We torque-limited the simulation using the stall torque *τ*_*s*_ = 3.5 Nm reported on the T-motor U8 datasheet^4^.

Since the simulated leg was modeled as a linear spring, we calculated the torques at the two opposing motors operating in parallel that were required to produce the forces observed at the foot, and then calculated the energy lost to heat. The electrical energy successfully converted to mechanical energy was modeled as $\dot{\theta }\tau$ for *θ* in Eq. 16 and *τ* the motor torque. The electrical energy lost to heat for our two motors operating in parallel was modeled as $2R_{m}{\left(\frac{c}{2}\right)}^{2}$ for *R*_*m*_ the resistance of the motor from the T-motor U8 datasheet and *c* the current that would be required from a single motor to produce the forces at the foot through the robot’s kinematics. This is an underestimation of the energetic losses, which at the very least include passive draw from the electronics. We also expect that the simulated losses should not exactly match the losses of the robot jumping on the physical media because the simulated granular media and physical media are different in grain size, density, friction, and so on. However, the approximate form of the force functions in the bulk-behavior model should be the same, so the simulations can still be used to generate hypotheses for the experiments in the physical world. For the plots in this paper, we added a constant to the estimated joules. We calculated this constant by comparing the actual estimated energetic cost of a jump with our physical system on granular media to a simulated jump with the same virtual extension stiffness and no active damping.

We ran simulations varying the foot size, the active damping coefficient, and the virtual extension stiffness of the robot’s leg to predict when the robot would be more likely to benefit from the active damping controller as compared with the compression-extension controller (Figure 2). To account for different ground types, we also scaled the simulated stiffness and damping forces from the ground. The effect of reaching the robot’s torque limit is evident in these simulations. Increasing the active damping coefficient initially increases the difference in the cost of a single jump relative to the compression-extension condition. However, after a certain point, the robot hits its torque limit more and more during its extension mode and gains no further benefit from increases to the active damping coefficient. There appears to be a trade-off with ground stiffness and foot size that affects the location of the “elbow” in the plot. The robot reaches the point of diminishing returns more quickly when the ground is less stiff and the foot is smaller.

A few more patterns emerge. In the compression-extension condition (active damping coefficient = 0), there is never a large difference between the joules cost for a smaller versus a larger foot. The maximum difference, in the softest ground condition with the stiff gain, was only 5 J. In contrast, when using active damping, the difference between the joules cost for different foot sizes with all other conditions held constant can be quite large. This is particularly the case when the ground is stiff and has little damping. When the ground stiffness is low and has larger damping forces, there is less of a difference in the joules cost for a single jump with different foot sizes. The greatest benefit from using active damping is conferred when the robot has a small foot and a high virtual extension stiffness.

Based on these simulations, we therefore make two predictions:

1) Jumps using the compression-extension controller with the same virtual extension stiffness should have similar energetic cost regardless of the size of the foot.
2) Jumps using active damping should have the highest savings when using the small foot and stiff extension spring coefficient, and the lowest savings when using the large foot and soft extension spring coefficient.

We compare the results of our physical experiments to these predictions in Section 3.

#### 2.3.2 Discrete Element Model Simulations

We used two discrete element model simulations performed with LAMMPS Improved for General Granular and Granular Heat Transfer Simulations (LIGGGHTS)^5^ an open-source discrete element modeling simulation tool, to compare the energy transferred to the ground from the compression-extension and active damping controllers. We generated two trajectories for a cylindrical foot moving through an idealized granular media using either the compression-extension or the active damping controller with our previously described simulation using the analytic force functions of the ground. These simulations can be seen in the Supplementary Video. The movements of individual grains underneath and around the robot’s foot were then simulated as it traveled along the two different trajectories^6^ The kinetic energy of the grains for the two different conditions is plotted in Figure 3. Under the compression-extension controller, the grains experienced a surge of kinetic energy when the robot enters its extension mode. In contrast, the active damping controller only imparted small amounts of kinetic energy to the ground over the course of its whole jump. These simulations provide additional corroboration for the empirical validity of the mathematical analysis in Section 2.2.2. The active damping controller reduces the energy transferred from the robot to the ground according to the bulk behavior model of the substrate mechanics.

### 2.4 Physical Experiments on Prepared Granular Media

In this section, we will describe the jumping experiments we performed on physical granular media. First we will describe the physical robot used in the jumping experiments. Next, we will describe the prepared granular media bed and how we controlled its preparation between experiments. We will then describe the experimental protocol we used for the jumps on physical media and how we calculated the quantities of interest, namely the energy consumption data and the jump heights. In these experiments we measured the actual energy consumption of the physical robot by measuring the mAh required to recharge the battery.

#### 2.4.1 Robot Used in Jumping Experiments

The robot we used in these experiments was a single-leg hopper using the same motor controllers and basic hardware as the Ghost Minitaur and T-motor brand U8 motors, but with custom control code to implement the classical compression-extension controller, and the active damping controller. The robot used two opposing motors to control the motion of its foot through a symmetric 4-bar linkage. We powered the robot with a 4-cell LiPo battery^7^ and charged it between experiments with a LiPo charger-balancer^8^.

**FIGURE 9:**
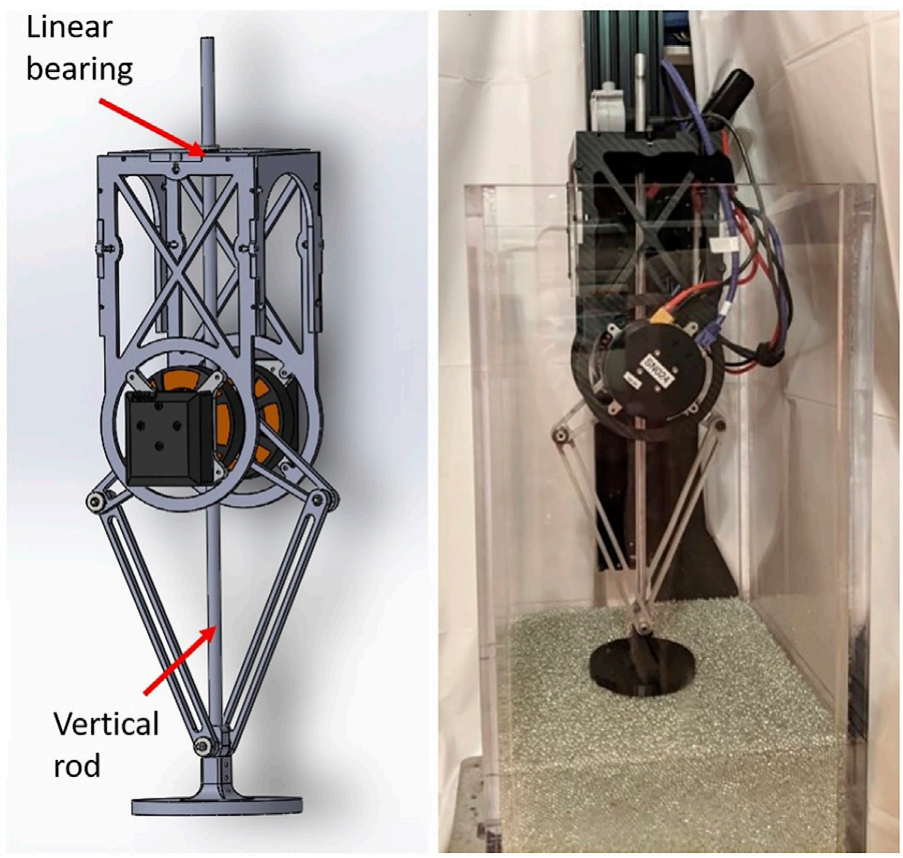
Left: The one-legged robot which was adapted for vertical jumping experiments on prepared granular media. Right: The full setup, with robot attached to linear rail in the sandbox. See Section 2.4.1 for more information.

The robot’s leg was modified to linearize the vertical motion of the foot (see Figure 9). The foot was attached to an aluminum rod which passes through a linear bearing housed in the 3D-printed top plate of the robot’s chassis, preventing any rotation of the foot as the motors move the foot vertically through the leg linkages. The rod was lubricated with machine oil at the start of each day that experiments were performed. The robot’s foot was 3D printed from ABS (acrylonitrile butadiene styrene, a thermoplastic polymer). Two foot sizes were used in these experiments: One with a radius of 0.051 m, and one with a radius of 0.038 m. The robot (Figures 6, 9) weighed 2 kg, accounting for the force exerted by the string potentiometer. The leg’s maximum length was 0.3 m and its minimum length, when fully compressed, was 0.1 m. The neutral length was set to 0.27 m. The robot can be seen performing a jump in the Supplementary Video. The robot was attached to a gantry plate and was free to move up and down along a vertical rail^9^ The angle of the vertical rail was checked using an analog angle gauge at least once per day of experiments.

The robot was programmed with a simple hybrid controller which cycled between two states: *compresssion*, in which the robot’s leg emulated a soft virtual spring through a low proportional and derivative gain on the motors through the leg kinematics (Figure 6), and *extension*, in which the leg emulated a stiff virtual leg spring. The robot switched from compression to extension when the leg was compressed beyond a small threshold, with a deflection of at least 0.05 m, and the rate of change of its leg length goes to zero, ${\dot{x}}_{b}-{\dot{x}}_{f}=0$ . The requirement that the robot’s leg must have deflected at least a small amount before the robot switches from compression to extension prevents the robot from switching to extension mode any time its leg length remains constant for some period of time, for example while the robot is in flight. The switch from extension back to compression occurs when the leg reaches its neutral leg length *l* (0.27 m for these experiments). The active damping controller added a force during extension, but did not otherwise change the control. The injection of energy caused by a sudden change from a soft to a stiff virtual leg spring is what causes the robot to jump (Figure 8).

We tested two virtual extension stiffnesses and five active damping gain conditions. We picked the active damping gains used in this study based on the suggestion from a previous study (Roberts and Koditschek, 2019) that high active damping gains would result in high energetic savings. The extension gains (300 and 400) were converted to stiffness coefficients (units: N/m) and the active damping gains (50, 100, 150, 200, and 250) were converted to damping coefficients (units: kg/s) using a least-squares fit we performed in a previous study on a similar leg (Roberts and Koditschek, 2019). For a stiffness coefficient *k*, stiffness gain *g*_*k*_, damping coefficient *d*, and damping gain *g*_*d*_, the formula for stiffness is *k* = 2.56*g*_*k*_ + 67.36, and the formula for the damping coefficient is *d* = 3.32*g*_*d*_ + 19.67. The plots in this paper use the fitted stiffness and damping coefficients to ease comparison with the simulations and enable other researchers to more easily extend our results to their own robot platforms.

#### 2.4.2 Controlled Granular Media Bed

The controlled granular media bed consisted of a clear acrylic box with a 0.3 × 0.3 m base and 0.6 m walls. We filled the box with 3.4 mm glass beads^10^ to a depth of 0.16 m. Before each jump, we lightly stirred the media and smoothed the surface. The surface smoothing was performed with a slider cut to the inside shape of the box from a sheet of thick cardboard. Wings at the top of the slider hooked over the top of the box as it was moved back and forth, ensuring that the bottom of the slider prepared the surface at the same depth for each jump. An example of granular media preparation can be seen in the Supplementary Video.

We tested the range of compactions that the 3.4 mm glass beads could occupy by pouring grains loosely into spherical flasks of five different diameters, filling the remaining space with water, and then compacting grains and removing water until no more grains could be added. Water volume measurements were collected for both the initially poured loose packed state and the final maximum compaction state. We then fit a model which estimates the compaction at the center of a spherical container filled with media as the percent of media in the boundary layer goes to zero for both the loose packed and hard packed states using the method described in (Miskin and Jaeger, 2014).

**FIGURE 10:**
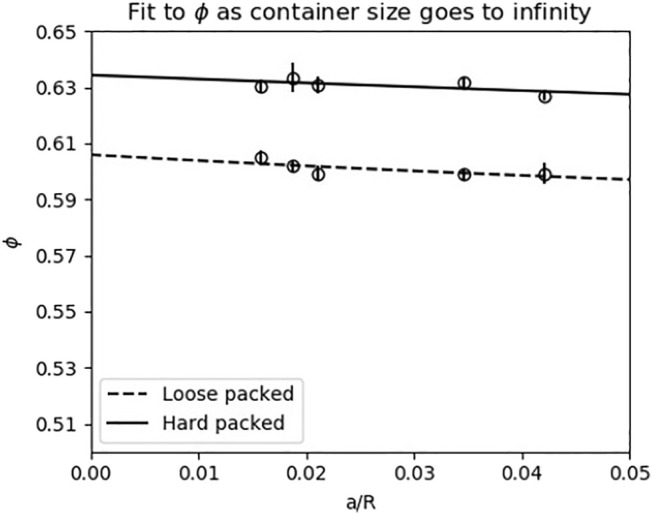
The difference between the minimum and maximum possible compactions *ϕ*, and therefore forces exerted by the ground, was 2*%*. The *x*-axis on this plot describes the ratio of the size of the grains (*a* = 3.4 mm) to the container radius. The *y*-axis corresponds to the volume fraction of the media, that is, and the percentage of space occupied by the grains. The y-intercepts on this plot therefore correspond to the volume fractions when the radius of the container goes to infinity. The range of the *y*-axis is determined by the possible range of granular media compactions. The vertical bars indicate one standard deviation. See Section 2.4.2 for more information.

The estimated volume fractions for the loose packed and hard packed states were 0.61 and 0.63, respectively (see Figure 10). To determine whether this variation was acceptable, we considered how the variation would affect the conclusions of our data. Since the maximum random close packing of a homogeneous granular media with spherical grains is about 0.64 (Cumberland and Crawford, 1987), in the 0.61–0.62 range, an impacting leg should not be able to substantially increase the compaction. Considering the force response from the granular media as a function of the compaction, the forces from the granular media should level off as the compaction approaches the maximum random closed packing. In Aguilar and Goldman (2016), the experimenters jumped a robot on a controlled granular media bed with a range of compactions. The expected relationship between compaction and jump height emerged, with the jump heights levelling off as the compaction exceeded 0.61. In the 0.61–0.63 range, the difference in jump heights is small and close to within experimental noise. This maximum 2% possible range of compactions in the granular media in our preparation was therefore deemed acceptable for our physical experiments.

Instead of testing on different preparations of granular media, which is typical for experiments on prepared granular media (Qian et al., 2015; Hubicki et al., 2016), we affected the force functions of the granular media by changing the robot’s foot size. The foot radius multiplies terms in all three of the ground’s force functions: The stiffness function *k*_*g*_ (*x*_*f*_), the damping function $d_{g}\left(x_{f}\right){\dot{x}}_{f}^{2}$ , and the added mass function *m*_*a*_ (*x*_*f*_). Changing the area of the foot, like changing grain properties such as friction or density, affects the overall force response from the ground.

It is worth noting that changing different parameters like foot size, grain density, and grain friction will all change the force response from the ground in slightly different ways. The friction of the grains appears in all three of the component granular media forces, while the grain density appears in only the added mass, and the dissipation functions. The foot area most directly affects the stiffness function of the foot, but it changes the depth at which the cone of added mass is fully formed under the robot’s foot, and therefore also affects the amount of added mass that can be recruited under the robot’s foot. Since the point at which the dissipation function’s coefficient becomes constant depends on the depth at which the cone is fully formed under the robot’s foot, this function is also affected by the foot size, but to a lesser extent. In short, no two parameters affect the model in exactly the same ways. For details on the granular media model used in these simulations and analysis, please see Aguilar and Goldman (2016).

#### 2.4.3 Experimental Protocol for Robot Jumping on Controlled Granular Media Bed

The full setup, with the robot constrained to a linear rail in the sandbox, is pictured in Figure 9. The robot’s foot was centered in the middle of the box to avoid boundary conditions during jumps, meaning that there was a minimum of about 0.1 m between the edge of the foot and any side wall during the experiment.

The energy consumption for the cost of transport measurements was calculated by recharging the battery after use and recording the mAh reported by the charger. The battery was recharged to the storage voltage of 15.4 V (3.85 V per battery) instead of the maximum voltage of 16.8 V because the relationship between percent of charge and voltage for lithium ion polymer batteries is nonlinear at the top of the voltage range (Jeon, 2014). Keeping the battery close to the storage voltage avoids this nonlinearity.

We took several additional precautions to reduce noise in the energy consumption measurements. First, if experiments had not been run the previous day, the battery was discharged by jumping the robot 20–50 times and then recharged before starting experiments for the day. This mitigated the effect of passive self-discharge. Second, we either randomized the order of experiments in each day or used block ordering, depending on how many experiments were to be conducted. Experiments were, however, performed in a rough ordering of foot size and extension gain: All experiments with the larger foot were performed first, and all experiments with the stiffer extension spring were performed first for a given foot size.

We also used a timer to ensure that all of the experiments took the same length of time (12 min). This was necessary because resetting the ground between jumps took significantly longer than the time the robot spent jumping. The passive power consumption of the robot’s electronics, even when the motors were not powered, and added significant noise to the energy cost measurements if this was not held constant. Sometimes, the motor controllers would report the incorrect leg configuration, and the robot would need to be restarted up to 3 times. When this happened, we quickly flipped the switch on and off, and noted how many starts were required. We were not able to detect a difference in the power consumption between trials with multiple starts versus one start.

We prepared and checked the experimental setup to ensure that it was consistent from day to day. Before starting experiments each day, we checked the angle of the robot’s vertical rail to make sure that it was within 1 degree of vertical. We added machine oil to lubricate the linearizing rod attached to the robot’s foot which passes through a linear bearing in the 3D-printed top of the robot’s chassis.

Each Experiment Proceeded as Follows

1) Connect battery to robot and turn the hard switch on, providing power to the robot’s electronics but only zero gain to the motors.
2) Start the timer.
3) Connect the robot’s microcontroller board to the laptop and extend and compress the leg to ensure that the robot is booted up and transferring data correctly.
4) Repeat steps 1 and 3 as many times as necessary to get accurate length readings.
5) Begin logging data to the laptop.
6) Remove the clamp that the robot is resting on and move the robot down until it is just past a mark on the string potentiometer indicating the start position (0.42 m).
7) Drop the robot.
8) Catch the robot before it touches down a second time.
9) Replace the clamp and allow the robot to rest while resetting the ground.
10) Repeat steps 7-9 for a total of 25 jumps.
11) Allow the robot to rest on the clamp until the full 12 min have passed.
12) Cut power to the battery and place it to charge. Stop logging data and upload the code for the next experiment.

We performed at least one control experiment per day, and almost always at least one from all of the same extension gains as the experiments with active damping that we tested that day. At least 10 experiments were performed for each combination of foot size, extension gain, and active damping gain, with more experiments performed for some control conditions.

#### 2.4.4 Calculation of Energy Consumption Data

During preliminary experiments (not reported), we found that a large source of variability was the amount of time that the robot spent passively drawing power from the battery while the experimenter reset the ground. Because the robot sometimes moved unexpectedly on startup or required several starts to appropriately link to its motors, the robot could not be fully disconnected from its power source while the ground was being reset. We were concerned that a stationary power supply might not sufficiently capture the transient high-current spikes, particularly during touchdown and the switch from compression to extension mode, that are of primary concern for dynamic legged locomotion. We also had extremely consistent data from a previous study in which we used the same power setup that we did here: The robot jumped a number of times with a battery, and then we recharged the battery and recorded the number of mAh reported on the charger (Roberts and Koditschek, 2019). We were therefore convinced that the method of measuring power consumption was sound, and the variability was coming from another source.

Measurements of the passive power consumption while the robot was hanging from a clamp for 20, 24, 30, 40, 50, and 60 min confirmed the hypothesis that passive power draw was a large source of variability in our initial experiments. Obtaining a linear fit to this data (*R* ^2^ = 1), we estimated that the passive power draw over a 12-min experiment added 86.2 to the mAh reported by the charger. We chose 12 min because this was more than sufficient time to consistently complete all 25 jumps and reset the ground adequately every time, even if there were several occasions when the ground needed extra time to be reset. The stance duration for a typical jump is less than 0.5 s and each experiment consisted of only 25 jumps, meaning that the robot spent only about 10 s in stance during each experiment. The time spent during stance was therefore considered trivial relative to the estimated passive power consumption, and 86.2 was subtracted from each measurement recorded from the charger.

#### 2.4.5 Calculation of Jump Height Data

The robot’s height during each jump was measured with a string potentiometer attached to the top of the robot, with its string attached to the top of the vertical rail the robot jumped up and down along. The data was captured by the robot’s controller board and passed to the experimenter’s laptop through a serial-to-USB connection.

After all experiments were complete, we used a custom script to display the logs and allow the experimenter to visually check each jump before accepting it as a data point. We rejected jumps where the log had a dropped packet or the script failed to correctly identify the apex of the jump. For a single condition, the minimum number of accepted jumps was 185 and the maximum number was 272.

## 3 Results

In general, the robot used more energy under the compression-extension controller than the active damping controller (Section 3.1). However, we did not see a relationship between the active damping coefficient and a reduction in the energetic cost to jump. A small foot appears to contribute to larger savings when using active damping than a larger foot (Section 3.2). Finally, there was no difference in the jump height when using active damping or the compression-extension controller (Section 3.4). All statistical tests reported in this section use a threshold of p < 0.05.

### 3.1 The Active Damping Controller Used Less Energy Than the Compression-Extension Controller to Jump

We used a one-way ANOVA to check for an overall relationship between active damping coefficient and energy consumption in the active damping controller, which was not significant at the p < 0.05 threshold for any combination of foot size and virtual extension stiffness (see Table 1 for a summary).

**TABLE 1:** This table contains the *p*-values for the ANOVAs testing whether there is a relationship between the coefficient used by the active damping controller and energy consumption.

Foot radius	Extension stiffness	*p*-value
0.04 m	850 N/m	0.366
0.04 m	1,100 N/m	0.411
0.05 m	850 N/m	0.472
0.05 m	1,100 N/m	0.294

Each row is the test of all active damping coefficients for that combination of leg extension stiffness and foot size. In all cases, there is no statistically significant relationship between active damping coefficient, and energy consumption.

We pooled all of the active damping conditions together and used a t-test to compare the pooled active damping condition to the control condition. There was a statistically significant difference between all of the pooled active damping conditions and their respective control conditions (p < 0.05) except for the large foot, soft spring condition (*p* = 0.071). See Table 2 for a summary. The simulations (Section 2.3.1) suggested that a smaller foot and a stiffer extension gain would lead to a larger difference between the active damping and the compression-extension controllers, which is consistent with our results. Pooling across active damping condition and foot size resulted in all comparisons having a statistically significant difference (p < 0.05). The data from the physical experiments is plotted in Figure 9.

**TABLE 2:** This table contains the *p*-values for t-tests comparing the pooled active damping controller conditions across each foot size and extension gain to the corresponding compression-extension conditions.

Foot radius	Extension stiffness	*p*-value
0.04 m	850 N/m	0.028
0.04 m	1,100 N/m	0.002
0.05 m	850 N/m	0.071
0.05 m	1,100 N/m	0.007

The simulations (Section 2.3.1) indicate that there is a point of diminishing returns for the active damping coefficient. Assuming that the model is correct, all of our physical experiments appear to have active damping coefficients beyond this point. Unfortunately, without collecting a large amount of data a priori, we were not able to determine ahead of time that all of our active damping gains would be above the point of diminishing returns.

We also performed individual statistical tests comparing each of the active damping conditions for a combination of foot size and extension gain. For each pairing of a control compression-extension condition and active damping condition, we first used an F-test to check for equal variances and then used a t-test to compare the conditions using the appropriate assumption about equal or unequal variances. Across all combinations of foot radius and virtual extension stiffness, the robot used less energy to jump with active damping than with the compression-extension controller. Most, but not all, of these comparisons were statistically significant (see Table 3).

**TABLE 3:** This table contains the *p*-values for all of the statistical tests performed with all combinations of foot size, leg extension stiffness, and active damping (AD) coefficient.

Foot radius	Extension stiffness	AD coefficient	*p*-value
0.04 m	850 N/m	186 kg/s	0.019
0.04 m	850 N/m	352 kg/s	0.081
0.04 m	850 N/m	518 kg/s	0.019
0.04 m	850 N/m	684 kg/s	0.025
0.04 m	850 N/m	850 kg/s	0.037
0.04 m	1,100 N/m	186 kg/s	0.041
0.04 m	1,100 N/m	352 kg/s	0.006
0.04 m	1,100 N/m	518 kg/s	0.001
0.04 m	1,100 N/m	684 kg/s	0.002
0.04 m	1,100 N/m	850 kg/s	0.002
0.05 m	850 N/m	186 kg/s	0.035
0.05 m	850 N/m	352 kg/s	0.113
0.05 m	850 N/m	518 kg/s	0.110
0.05 m	850 N/m	684 kg/s	0.128
0.05 m	850 N/m	850 kg/s	0.051
0.05 m	1,100 N/m	186 kg/s	0.007
0.05 m	1,100 N/m	352 kg/s	0.028
0.05 m	1,100 N/m	518 kg/s	0.103
0.05 m	1,100 N/m	684 kg/s	0.003
0.05 m	1,100 N/m	850 kg/s	0.002

### 3.2 Energy Saved From Using Active Damping With Different Foot Sizes and Leg Extension Stiffnesses

We calculated the energy saved by using active damping relative to the compression-extension controller, using data pooled across active damping conditions. Recall that this pooling is justified by the lack of statistically significant difference between any two active damping coefficient conditions for a combination of foot size and leg extension stiffness (Section 3.1). Calling CE the average cost of a single jump under a compression-extension controller with a certain foot size and extension stiffness and AD the average cost of a single jump under the active damping controller for that same foot size and extension stiffness, the percent savings afforded by the active damping controller is (CE-AD)/CE. The highest percent savings, 15%, was in the small foot, stiff extension spring condition, and as expected from the simulation results. The next highest was in the small foot, soft extension spring condition (14*%*), followed by the large foot, stiff spring condtiion (11%). The soft spring, large foot condition saved the least energy (10%). These results are summarized in Table 4. We can thus see that the percent savings was much more affected by the foot size than the extension stiffness. The data suggest that there is an appreciable increase in the percent savings when using a smaller foot, but we cannot conclude from this data that the leg extension stiffness has a non-negligible effect on the percent savings conferred by using active damping.

**TABLE 4:** The smaller foot, higher stiffness condition had the largest savings when using active damping. Here, the “percent savings” was calculated for a foot radius and stiffness coefficient in the following manner: (CE-AD)/CE for CE the average joules required for the robot to jump once using the compression-extension controller, and AD the average joules required for the robot to jump once using the active damping controller, with experiments pooled across all active damping gains.

Foot radius	Extension stiffness	Percent savings (%)
0.04 m	850 N/m	14
0.04 m	1,100 N/m	15
0.05 m	850 N/m	10
0.05 m	1,100 N/m	11

### 3.3 Comparison of Results From Physical Experiments to Predictions From the Simulations

From the simulations (see Figure 2; Section 2.3.1), we expected two things. First, we expected that there should be a negligible difference between the joules per jump for a robot jumping with the compression-extension controller using the same virtual extension stiffness with different sized feet. Second, we expected that there should be a larger reduction in the joules per jump for the same active damping coefficient when using a smaller foot. These patterns are not refuted by the data. Using the same method of first testing for equal variance with an F-test and then performing a t-test, we found that differences between the joules per jump for the same virtual extension stiffness but different foot sizes were not statistically significant for the compression-extension condition (p > 0.5 for both). However, seven of the ten extension stiffness-active damping pairs were statistically significant (p < 0.05). One of the three which were not statistically significant was from the smallest active damping coefficient with the stiffer leg (*p* = 0.245) and the other two were from the softer spring condition (active damping gain = 100, *p* = 0.079; active damping gain = 250, *p* = 0.057). Comparing the larger and smaller foot sizes for the pooled active damping condition, both the stiffer and softer extension springs had a significant difference for joules per jump with foot size (p < 0.000 1 for both virtual extension stiffnesses). See Table 5 for a summary.

**TABLE 5:** There was a statistically significant difference between the cost of a single jump when using different foot sizes for 7/10 combinations of active damping (AD) coefficient and leg extension stiffness.

Extension stiffness	AD coefficient	*p*-value
850 N/m	186 kg/s	0.043
850 N/m	352 kg/s	0.079
850 N/m	518 kg/s	< 0.001
850 N/m	684 kg/s	0.017
850 N/m	850 kg/s	0.057
1,100 N/m	186 kg/s	0.245
1,100 N/m	352 kg/s	0.010
1,100 N/m	518 kg/s	0.003
1,100 N/m	684 kg/s	0.005
1,100 N/m	850 kg/s	0.028
850 N/m	Pooled	< 0.001
1,100 N/m	Pooled	< 0.001

When all of the active damping gain conditions were pooled for a combination of foot size and extension gain, the findings were statistically significant in both cases.

### 3.4 Jump Heights on Physical Granular Media

Previous simulations and experiments (Roberts and Koditschek, 2018; Roberts and Koditschek, 2019) suggested that the robot should jump slightly less high when using the active damping controller than when using the compression-extension controller, with a maximum height loss of less than 5*%*. We did not see that loss in these experiments (see Figure 10). Repeating our procedure of performing an F-test for equal variance and then a t-test with the appropriate assumptions, we found either a statistically significant *increase* in jump height when using active damping compared with the compression-extension controller, and or no statistically significant difference. This means that the robot is either jumping to the same height when using active damping and the compression-extension controller, or—if there is a difference—the difference favors the active damping controller. In other words, there is absolutely no loss to jump height from using active damping. Whereas previous work had suggested that the active damping controller came at a slight cost of a loss to jump height, these experiments on physical media suggest that this is not always the case. That said, the largest increase in jump height measured was only 3 mm. Although the difference in height was statistically significant, it is small enough to be negligible.

## 4 Discussion

### 4.1 Application to Geoscience Research

Minitaur’s direct-drive legs can also be used as force sensors. Because there are no gearboxes on the legs, deflections at the foot can be sensed more transparently than possible through an intermediating transmission (Kenneally et al., 2018). When the forces exerted by the leg for a given deflection are known, the deflection can be used as an effective force sensor. By dragging a single leg across the surface of a patch of desert sand or poking the leg straight down into the ground, it is possible to measure ground properties such as shear stress and stiffness which are relevant to erodibility (Qian et al., 2017). These represent quantities of significant interest for researchers interested in studying natural erosion processes like desertification (Roberts et al., 2014a; Roberts et al., 2014b). Accurate measurements of these forces require that the robot have small, lightweight feet, and no gearboxes.

We regularly take our robots on field missions with geoscientist collaborators who study deserts and other challenging environments like forested hillslopes (Qian et al., 2017). Our larger aim is to outfit our collaborators with a heterogeneous team of robots: One or more RHex robots (Haynes et al., 2012), which are heavily geared 10 kg legged robots with a broad flat back that can be used to carry a large sensor payload, and one or more Minitaurs. The Minitaurs should run ahead and scout for locations of interest by performing simple ground intrusion and shearing tests, while the RHex robots slowly carry payloads of sensors to the most interesting locations, and communicate data to the scientists as they are captured. The semi-autonomy of a team of robots working directly under human supervision provides a bridge between the current methods used to perform planetary science on Earth and the research modes used on planets such as Mars, where human interaction with the rovers is very slow.

However, the Minitaur robot is currently challenged by long-distance locomotion in the desert. While it is able to perform short runs, the motors quickly overheat, and the robot cannot yet be relied on as a self-transporting force sensor. Measurements using the Minitaur hardware and software are currently conducted with a single leg strapped to the back of the heavily geared RHex robot, leaving little room for an additional sensor payload, and removing the possibility of having two independently behaving robots (Qian et al., 2017). To realize this heterogeneous team, it is necessary to improve the energetic efficiency of the Minitaur robot on granular media such as dry desert sand without appreciably changing the robot’s physical architecture.

### 4.2 Strengths and Contextualization of the Present Work

In this work, we demonstrated that the active damping controller reduces the energetic cost of transport without reducing the robot’s jump height, a proxy for the effectiveness of locomotion. We demonstrated this with a combination of simulations, analysis, and experiments in physical granular media. In other words, when using active damping, the number of joules required to take a single jump was significantly reduced without any loss to the height of the jump. Our data also suggest the general trend that a robot using a smaller foot will benefit more from using active damping than a robot with a larger foot.

This method of reducing energy cost, because it uses basic principles of the energetic properties of sand, is more similar to adaptations like increasing the foot size than to adaptations like optimizing a controller for a single jump based on a model of a particular ground. As a result, this method may provide benefit in comparison to optimization and reinforcement learning based control methods because the robot does not need any awareness of the ground or its properties. The robot only needs to know the intrusion velocity of its foot. The robot does not even need to know whether it is on sand or rigid ground, because if the intrusion velocity is zero, the active damping force will be zero, and the robot will jump with its nominal compression-extension controller, which is effective on rigid ground.

In contrast to methods like the one presented here, methods relying on models of the environment may be more effective in specific conditions where there is data available, or where the ground conditions are relatively consistent (for example, Chang et al. (2019)). However, this method should reliably reduce the energy cost of jumping in any previously unencountered situation where the ground exerts bulk behavior forces like a granular media. Since variation can occur quickly in natural environments, a robot locomoting in a natural desert will likely need to adapt within one or two steps to changes in the ground stiffness. Building an accurate and useful representation of a truly unknown environment is a very difficult task which we suggest is not necessary for many basic locomotion behaviors (Roberts et al., 2020).

Since the active damping controller provides more benefit with smaller foot sizes, it will be most effective when the robot’s feet cannot be significantly enlarged. For example, if the robot needs to walk over a mixed terrain with both sandy portions and rocky portions that would be more easily traversed with a smaller foot that can hook into crevices and lift over rocks without getting stuck, it might be more useful to use active damping than to increase the foot size. For our application, in which the robot’s foot size cannot be significantly increased without reducing the utility of its feet as force sensors, the active damping controller is also an approach well suited to increasing endurance without diminishing capability.

The results in this paper also warrant a further discussion about the previous results from Roberts and Koditschek (2019), in which we jumped the same physical robot on top of a lightly geared robot emulating the forces from a compressible ground. In that work, we found that a higher active damping coefficient relative to the virtual extension stiffness resulted in a higher energy savings, which we did not replicate in these results on physical granular media, finding instead that varying the active damping coefficient did not change the energetic savings. The most likely explanation is that the robot’s motors are hitting their torque limits during the extension mode in our experiments on physical granular media, meaning that there is very little difference from the motor’s point of view between the different active damping coefficients. As we saw in the simulations for this paper (Section 2.3.1; Figure 2), when we reach the artificially set torque limits, there is no further benefit from increasing the active damping coefficient after a certain point. This is further supported by the fact that the Ghost motor controllers used on our robot have a safety limit in their firmware, meaning that there is a sudden hard limit on the torque output from the motor, and the physical motors should behave similarly to the simulated motors. In Roberts and Koditschek (2019), the most significant increases from increasing the active damping gains were in the low virtual extension stiffness conditions, when a higher damping gain would not cause the robot to reach a torque limit. We were not able to test those low extension stiffness conditions on the physical setup due to the softness of the granular material and the necessity of the experimenter to be able to catch the robot while in flight. In the minimum stiffness, minimum foot size condition tested in this paper, and the robot’s foot achieved a height of only about 0.02 m from the undeflected surface of the ground.

### 4.3 Limitations of the Present Work

One limitation of this work is that we only consider flat granular media being penetrated vertically. We do not consider the role of shearing forces or inclination in the granular media force responses. Shearing is an important part of locomotion, and more work in granular media physics is required to accurately model the forces in response to concurrent shearing and vertical intrusion. Work on inclined granular media (Forterre and Pouliquen, 2008; Han et al., 2019) suggests that the force response is similar in form but reduced in comparison to the force response of flat media. However, in neither case are the forces sufficiently well understood to perform the same sort of analytic simulations and modeling used in this paper.

Another limitation of the present work is that we could not see an effect of changing the active damping coefficient, likely due to all of the leg extension stiffnesses and active damping coefficients being sufficiently large that the robot hit its torque limits during the experiments. This could be addressed in future work by using a “stiffer” granular media, which would make it possible to use lower extension stiffnesses, and lower active damping gains. In this study we did not characterize the “stiffness” and “damping” functions for the ground. Such characterization would have made it easier to determine appropriate stiffness and damping functions to use in simulation. Some of the discrepancies between our observations in the simulations and the physical world could be attributed to this lack of information about the exact values of the stiffness and damping functions of the ground. Another option would be to use a previously characterized granular media which requires a fluidized bed.

Finally, we only performed a limited number of experiments, and cannot draw conclusions about the effects of varying the leg extension stiffness, the active damping gain, or the properties of the granular media from our physical experiments. Without more experiments in different active damping gain conditions, it is impossible to determine the relationship between active damping gain, and the reduction in energetic cost. Even without using a fluidizing bed, future work could include using grains with different densities or surface frictions to change the stiffness, and damping functions of the ground. Testing on more than one physical media would strengthen the claims that using active damping mitigates the energy lost to the ground during jumping. Also, without more experiments in different conditions generally, it is impossible to optimize the savings from the active damping controller in comparison to the compression-extension controller. By testing more combinations of foot sizes, active damping gains, extension stiffnesses, and characterized granular media types, it would be possible to make claims about the direct relationships between these variables. The conclusion that we can draw from our physical experiments are limited to the fact that active damping generally decreases the cost of a single jump to a certain height, and that smaller feet increase the savings conferred by active damping in comparison to the compression-extension controller.

### 4.4 Summary and Potential Future Directions

In our physical experiments, we found a consistent reduction in the energetic cost of performing a single jump when using active damping in comparison to the base compression-extension controller. This reduction in the power drawn from the battery did not result in a reduction in jump height, implying that the active damping controller for locomotion on sand uses the energy from the battery more efficiently for the goal of locomotion. We conclude that the active damping controller saves significant energy in comparison to the base compression-extension controller.

Future work in this area could extend these results by performing physical experiments with more granular media types, particularly media that would be more like the media that a robot would encounter on a sand dune. With the use of a fluidizing bed, it would be possible to test media with a much smaller diameter. Media with different densities and surface frictions would also be particularly interesting to test, as both of these parameters would increase the overall force response from the ground and enable the experimenters to test with smaller feet and softer leg extension spring coefficients. Changing the relative humidity would be another interesting avenue to explore along this dimension, as this would make it possible to extend the results beyond dry granular media. It would also be interesting to perform experiments with angled intrusions and on tilted granular media. Once we understand how the performance of the controller is affected by shearing and tilting, the active damping controller should be compared with the compression-extension controller on a freely behaving robot in a natural desert environment.

The active damping controller could also potentially be composed with other controllers for locomotion. For example, one controller typically used on the Minitaur robot uses negative damping to inject energy into the virtual leg spring during stance (De and Koditschek, 2018). Positive damping, meaning a positive damping coefficient on the virtual leg spring, slows down how quickly the leg extends. In contrast, negative damping makes the leg move *faster*, which causes the robot to jump in a visually similar way to the controller we use in this study, which injects energy by changing the leg stiffness. However, since negative damping increases the speed of motion of the leg, on granular media such a controller would cause the robot’s foot to push very quickly into the sand, potentially transferring a large amount of energy to the ground quickly. Furthermore, if we think of the two controllers acting in parallel, one controller will be adding negative damping to the leg to energize it while the other controller adds positive damping to slow down the foot’s intrusion with the ground. In order for the robot to jump successfully, the controllers would need to be carefully balanced, and which might seriously limit the range of ground conditions on which the robot could jump. In order to test the general utility of the active damping controller on sand, it should be possible however to compose it with controllers developed using optimal control or machine learning techniques.

## Data Availability

The raw data supporting the conclusion of this article will be made available by the authors, without undue reservation.

## Footnotes

1 RFT models the force response using multiple experimentally determined and measured parameters about the individual grains making up the granular media. It should be noted that a more recent model captures the same force response prediction using only the internal friction angle in a single universally scalable model (Kang et al., 2018).

2 Ghost Robotics, 3,401 Gy Ferry Ave, Philadelphia, PA 19146.

3 Please see https://github.com/KodlabPenn/jumping_on_granular_media/blob/main/README.md for source code.

4
https://store.tmotor.com/goods.php?id=322.

5
https://www.cfdem.com/liggghts-open-source-discrete-element-method-particle-simulation-code.

6 Thanks to Swapnil Pravin, Temple University, for running these pre-generated trajectories through LAMMPS.

7 ThunderPowerRC, 3,550 East Post Road Suite 500 Las Vegas, NV 89 120, Reaper series, and part number TP6600-4SR70.

8 ThunderPowerRC, part number TP610HVC.

9 OpenBuilds, 719 Whig Ln, Monroeville, and NJ, 08343.

10 Jaygo, Inc., 7 Emery Ave, Randolph, and NJ, 07869.
